# Omics-driven research progress on phosphate activation mechanisms and stress adaptability regulation of Phosphate-Solubilizing Microorganisms (PSMs)

**DOI:** 10.3389/fmicb.2026.1758069

**Published:** 2026-02-18

**Authors:** Yan Zhang, Chengyi Zou, Caiming Gou, Jia Li, Xiaohua Li, Bo Chen

**Affiliations:** 1 School of Agriculture, Forestry and Food Engineering, Yibin University, Yibin, China; 2 Solid-State Fermentation Resource Utilization Key Laboratory of Sichuan Province, Yibin University, Yibin, China; 3 Sichuan Yibin Yiquan Wine Co., Ltd., Yibin, China

**Keywords:** multi-omics, phosphate activation mechanisms, Phosphate-Solubilizing Micro-organisms (PSMs), stress adaptability, sustainable agriculture

## Abstract

Phosphorus (P) is an indispensable macronutrient for crop growth and development, but most phosphorus in soil exists in insoluble forms with extremely low availability. Although the application of traditional chemical phosphorus fertilizers can meet the phosphorus demand of crop growth, the extensive exploitation of phosphate rock resources has led to problems such as phosphate rock depletion and environmental pollution, highlighting an urgent need for sustainable phosphorus management strategies. Phosphate-Solubilizing Microorganisms (PSMs) provide an environmentally friendly biological approach to address this challenge. Existing reviews mainly focus on the basic phosphate-solubilizing mechanisms and agricultural applications of PSMs, but lack integration of cutting-edge directions such as omics-based mechanism analysis, stress adaptability regulation, and compound microbial inoculant design. From the innovative perspective of “omics-driven mechanism analysis - stress adaptability regulation - multifunctional inoculant development,” this review systematically summarizes: (1) the distribution characteristics of PSMs and environmental adaptability differences among functional groups; (2) the molecular regulatory networks of core phosphate activation mechanisms (acidification, organic acid secretion, phosphatase production) based on multi-omics, with a focus on comparing mechanism-specificity between bacteria and fungi; (3) the regulatory rules and adaptive mechanisms of PSMs activity under stress factors such as pH, heavy metals, and salinity; (4) the host-specific interaction mechanisms between PSMs and plants, as well as the regulatory effects on rhizosphere microenvironment; (5) the formulation development, field application bottlenecks, and large-scale promotion strategies of PSMs biofertilizers. Finally, the current research limitations (e.g., fragmented molecular mechanisms, significant differences between field and laboratory effects) are critically analyzed, and future research directions are proposed, including integrated multi-omics analysis, breeding of high-efficiency stress-tolerant strains, and long-term field validation. By integrating cutting-edge molecular mechanisms and practical application bottlenecks, this review provides a novel theoretical framework for the precise development and sustainable agricultural application of PSMs, which is of great significance for promoting the construction of an eco-friendly agricultural system.

## 1 Introduction

Phosphorus, as a core macronutrient for crop growth and development, is involved in key physiological processes such as nucleic acid synthesis, energy metabolism (ATP), and photosynthesis (Kaur et al., 2024; Rawat et al., 2020; Somtrakoon and Chouychai, 2021). Although the total soil phosphorus content is abundant, more than 95% exists in inorganic insoluble forms (e.g., calcium phosphate, iron phosphate) and organic phosphorus (e.g., phytic acid, nucleic acids) that are difficult for plants to absorb. Insufficient supply of available phosphorus has become a key bottleneck restricting the improvement of crop yield (Chakraborti et al., 2025; Fatima et al., 2020; Khan et al., 2007). In alkaline soils, phosphorus is prone to combine with calcium to form insoluble salts, while in acidic soils, it precipitates with iron and aluminum, further reducing phosphorus availability (Mahdi, 2012; Naorem et al., 2023); organic phosphorus needs to be converted into inorganic phosphorus through mineralization before being absorbed by plants, and the natural mineralization rate is extremely low (Billah et al., 2019; Fu et al., 2024; Haile et al., 2016; Khan et al., 2009). To compensate for phosphorus deficiency, traditional agriculture relies heavily on the application of chemical phosphorus fertilizers, but their utilization rate is less than 20%. The remaining phosphorus is fixed by soil, which not only causes the depletion of phosphate rock resources but also triggers ecological problems such as soil degradation and water eutrophication (Fatima et al., 2021; Timofeeva et al., 2022). Therefore, developing environmentally friendly and cost-effective phosphorus activation technologies is a core demand for achieving sustainable agricultural development (Kumar et al., 2025; Silva et al., 2023).

Phosphate-Solubilizing Microorganisms (PSMs) include bacteria, fungi, actinomycetes and other groups. They convert insoluble phosphorus into plant-available forms through various mechanisms, significantly reducing the dependence on chemical phosphorus fertilizers, which is consistent with the core demands of the United Nations Sustainable Development Goals (SDGs) such as “Zero Hunger” and “Climate Action” (Ishfaq et al., 2023; Pfeifer et al., 2024). In recent years, a large number of reviews on PSMs have been carried out. For example, Pang et al. (2024) systematically elaborated the process of soil phosphorus transformation and plant uptake driven by PSMs, but lacked in-depth analysis of molecular mechanisms (Fatima et al., 2020; Naorem et al., 2023) focused on the application of PSMs in rice systems, with a relatively single research scenario (Chakraborti et al., 2025). Current reviews generally have three deficiencies: first, they tend to repeat basic phosphate-solubilizing mechanisms and lack integration of molecular regulatory networks based on omics technologies; second, they insufficiently discuss the adaptive mechanisms of PSMs under stress environments (heavy metals, salinity-alkalinity, drought); third, they lack critical analysis of practical bottlenecks such as biofertilizer formulation development and field effect stability. Based on this, this review takes “omics analysis - stress adaptation - practical application” as the core thread, integrates recent multi-omics research results, critically analyzes the environmental dependence of PSMs phosphorus activation mechanisms, deeply discusses cutting-edge application bottlenecks and solutions, clarifies the differentiated innovation points between this review and existing ones, and provides more targeted theoretical support for the efficient development and agricultural application of PSMs.

## 2 Distribution characteristics and functional group differentiation of PSMs

Phosphate-Solubilizing Microorganisms are widely distributed in various ecological environments such as agricultural soils, saline-alkaline soils, mine tailings, and forest soils. Their community composition and activity are regulated by factors such as soil texture, tillage methods, and nutrient levels (Cheng et al., 2023; Wu et al., 2019; Xiao et al., 2022; Xu and Chen, 2025; Xu et al., 2021). For example, in the saline-alkaline gradient soils of western Songnen Plain, the community assembly of PSMs is mainly driven by alkalization degree and salinity (Xu et al., 2021); under different fertilization methods, the abundance of active PSMs in soil varies significantly, and the application of organic fertilizers can significantly enhance the in-situ activity of PSMs (Li et al., 2022; Li H. Z. et al., 2024). This wide distribution and environmental adaptability provide a resource basis for the screening and application of PSMs in different ecological scenarios.

The functional groups of PSMs are mainly bacteria and fungi, while the research on actinomycetes is relatively weak. There are significant differences in phosphorus-solubilizing ability and environmental adaptability among the three types of microorganisms (Table 1).

**TABLE 1:** Functional characteristics and research gaps of three types of Phosphate-Solubilizing Microorganisms.

Functional groups	Core representative genera	Dominant Phosphate-Solubilizing Mechanisms	Environmental adaptability characteristics	Research gaps
Phosphate-solubilizing actinomycetes	*Streptomyces, Francisella* (Lopez et al., 2025)	Synergistic effect of organic acids and phosphatases (Iftikhar et al., 2024; Li et al., 2023; Wang et al., 2023)	Strong tolerance to poor nutrition; potential advantages in degraded soils	Insufficient resource screening; very limited research on phosphate-solubilizing molecular mechanisms
Phosphate-solubilizing fungi (psf)	*Aspergillus, Penicillium, Penicillium* (Alori et al., 2017; Boubekri et al., 2023)	Secrete high concentrations of citric acid and oxalic acid; strong chelating capacity; hyphae can penetrate soil pores (Lee et al., 2012; Lobo et al., 2019; Wang et al., 2023)	Higher phosphate-solubilizing efficiency than bacteria in acidic soils; stronger low-temperature tolerance	Lagging genomic analysis; unclear interaction mechanisms with bacteria in compound microbial inoculants
Phosphate-solubilizing bacteria (PSB)	*Bacillus, Pseudomonas, Enterobacter* (Das Mohapatra et al., 2024; Jiang et al., 2020; Vasques et al., 2024)	Secrete organic acids such as gluconic acid and lactic acid; high phytase activity; some strains produce siderophores (Bashir et al., 2024; Lee et al., 2012; Lobo et al., 2019; Wang et al., 2023; Wang X. et al., 2025; Zhu Y.-G. et al., 2024)	Rapid reproduction, easy to cultivate; obvious advantages in neutral/weakly alkaline soils; some strains have heavy metal/saline- alkali tolerance	Unclear host-specific interaction mechanisms; poor stability of long-term stress adaptability

### 2.1 Phosphate-solubilizing bacteria (PSB)

Phosphate-solubilizing bacteria are the most fully studied functional group, among which *Bacillus* and *Pseudomonas* are the two groups with the greatest application potential. *Bacillus* (e.g., *Bacillus megaterium*, *Bacillus subtilis*) efficiently dissolves inorganic phosphorus such as calcium phosphate by secreting various organic acids and phytases, and simultaneously produces ACC deaminase and plant hormones, possessing both growth-promoting and stress-tolerant functions (Thampi et al., 2023; Wang et al., 2023); *Pseudomonas* (e.g., *Pseudomonas aeruginosa*) exhibits excellent performance in promoting crop growth and phosphorus uptake through the synergistic effect of organic acids, siderophores, and plant growth regulators, and its application value has been confirmed in crops such as peanuts and wheat (Anonymous, 2024; Wang et al., 2023). Some strains of *Enterobacter* also have the potential to remediate heavy metal-contaminated soils by regulating rhizosphere phosphorus cycling to reduce the bioavailability of heavy metals (Bashir et al., 2024; Iftikhar et al., 2024; Li et al., 2023; Wang X. et al., 2025).

### 2.2 Phosphate-solubilizing fungi (PSF)

Phosphate-solubilizing fungi are the core functional group for phosphorus activation in acidic soils. Their phosphorus-solubilizing advantages stem from two aspects: first, they secrete high-concentration organic acids with strong chelating ability (e.g., oxalic acid, citric acid), which can directly act on the surface of phosphorus minerals (Lu et al., 2025; Šeremešić et al., 2024); second, mycelia can penetrate small soil pores, contact phosphorus mineral particles that are difficult for bacteria to reach, and expand the scope of action (Šeremešić et al., 2024). For example, when *Penicillium chrysogenum* strain PSF-4 dissolves tricalcium phosphate and iron phosphate, the concentration of soluble phosphorus is significantly positively correlated with the secretion of carboxylic acids, and the phosphorus-solubilizing efficiency in acidic soils is 2–3 times that of bacteria (Jiang et al., 2020; Lu et al., 2025). Some *Trichoderma* strains also have biocontrol functions, which can inhibit the growth of plant pathogens and achieve a synergistic effect of “phosphorus solubilization+disease prevention” (Wang et al., 2023).

### 2.3 Phosphate-solubilizing actinomycetes

Actinomycetes are a group with insufficient resource exploration. Existing studies have confirmed that *Streptomyces* and *Francisella* have phosphorus-solubilizing ability, which activates phosphorus through the synergy of organic acids and phosphatases (Iftikhar et al., 2024; Wang et al., 2023). Due to their strong tolerance to poor nutrition and stress, actinomycetes have unique potential in the ecological remediation of extreme soils such as mine tailings and degraded forestlands. However, current research only stays at the strain screening level, and molecular mechanisms and application effect verification are relatively weak.

## 3 Omics-based analysis of molecular mechanisms of PSMs phosphorus activation

**FIGURE 1:**
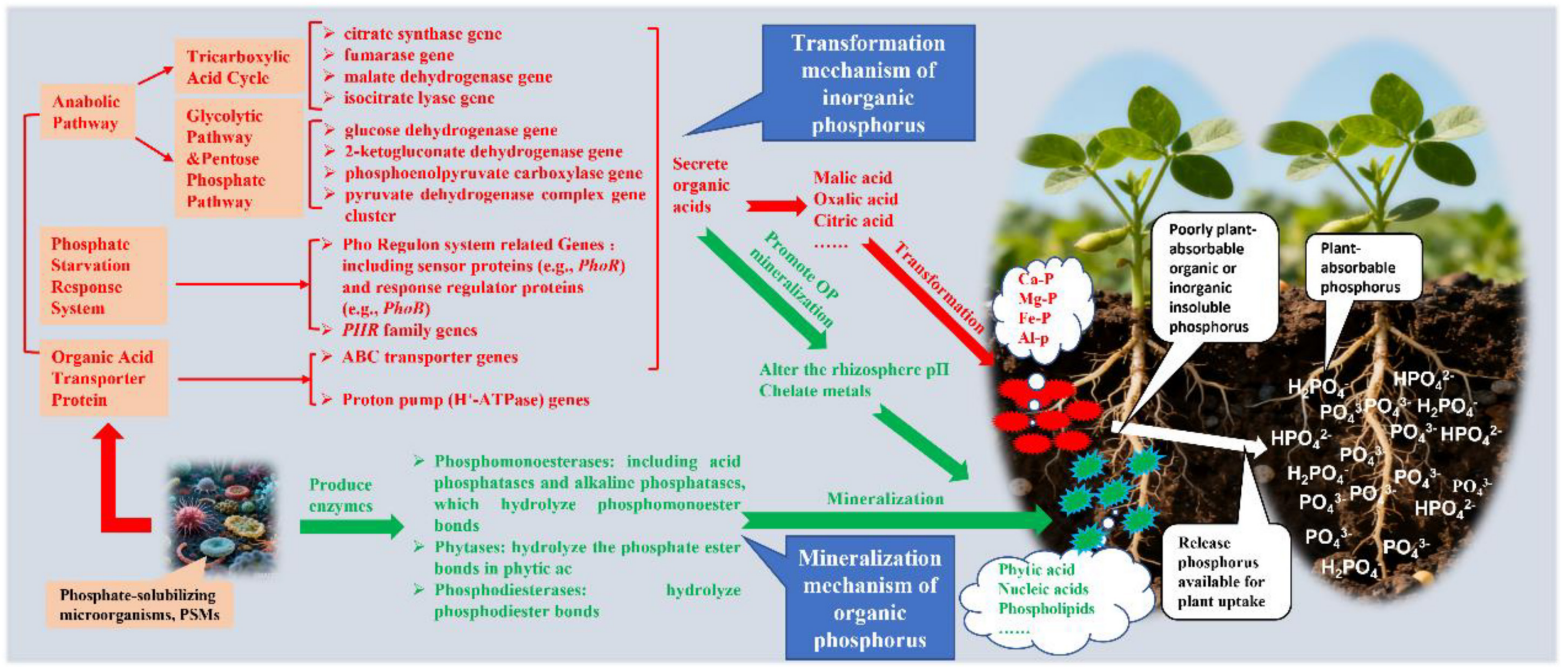
Mechanism of Phosphate-Solubilizing Microorganisms (PSM) solubilizing insoluble phosphorus (Liu et al., 2023; Salsabila et al., 2023; Sivasakthivelan et al., 2021; Zeng et al., 2024; Zhu Y. et al., 2024)

The phosphorus activation mechanisms of PSMs are centered on “organic acid secretion” and “phosphatase production,” supplemented by acidification and secretion of growth-promoting compounds. Traditional studies mainly focus on mechanism phenotypes (Figure 1). In recent years, the application of omics technologies (genomics, transcriptomics, metabolomics) has revealed the complexity of their molecular regulatory networks, providing targets for the breeding of high-efficiency strains.

### 3.1 Molecular regulatory network of organic acid secretion

The synthesis and secretion of organic acids are the core pathways for PSMs to dissolve inorganic phosphorus. Their molecular regulation involves the synergistic effect of carbon metabolism pathway genes, phosphorus starvation response systems, and transporter protein genes (de Almeida Leite et al., 2024; Lü et al., 2011; Rodríguez and Fraga, 1999). Genomic studies have shown that PSMs generally carry key enzyme genes of glycolysis and tricarboxylic acid (TCA) cycle, such as glucose dehydrogenase (gcd) gene of *Pseudomonas* and citrate synthase gene of *Bacillus*. The expression of these genes directly determines the type and yield of organic acids (Campo and San Segundo, 2020; Sharon et al., 2016; Sharma et al., 2021). For example, under low phosphorus conditions, TCA cycle-related genes (e.g., malate dehydrogenase gene) in *Pseudomonas aeruginosa* are significantly upregulated, driving the increase in the secretion of citric acid and malic acid (Wang et al., 2022); while the expression of gcd gene in *Pseudomonas* strain JW-SD2 is regulated by environmental phosphorus concentration. Under low phosphorus conditions, the expression level increases by 3.2 times, accompanied by the increase in gluconic acid secretion and medium acidification (Su L. et al., 2023; Wang Y. et al., 2025; Zeng et al., 2015).

The phosphorus starvation response system (e.g., Pho Regulon system in bacteria) is the core regulatory hub for organic acid secretion. Under low phosphorus conditions, the sensor protein PhoR is phosphorylated to activate the response regulator protein PhoB, which binds to the promoter region of organic acid synthesis genes to upregulate gene expression (Sharon et al., 2016). It is worth noting that the regulatory networks of different PSMs have species specificity: the regulation of organic acid secretion in fungi may depend on PHR family transcription factors, which have evolutionary differentiation from the Pho Regulon system in bacteria (Gurbanov et al., 2021; Li et al., 2025); while the relevant regulatory mechanisms of actinomycetes have not been reported, which is a key gap for future research.

The extracellular secretion of organic acids depends on transporter protein genes, such as ABC transporter and proton pump (H^+^-ATPase) genes (Amy et al., 2022). The expression of H^+^-ATPase genes not only provides power for the efflux of organic acids but also directly acidifies the rhizosphere environment, enhancing phosphorus dissolution efficiency (Amy et al., 2022). Currently, only a few transporter protein genes have been identified, and their substrate specificity (e.g., transport preference for different organic acids) is not clear, which limits the efficiency of enhancing organic acid secretion through genetic engineering.

### 3.2 Molecular regulatory mechanisms of phosphatase production

Phosphatases (phytases, phosphomonoesterases) are key enzymes for PSMs to mineralize organic phosphorus, and their expression is also regulated by the phosphorus starvation response system. Under low phosphorus conditions, the phosphorus starvation response genes (e.g., phoD, phoA) of PSMs are activated, driving the synthesis and secretion of phosphatases (Chen et al., 2025; da Silva Rodrigues et al., 2024; Gao et al., 2025; Yang et al., 2022). For example, in phoD gene-harboring microorganisms in karst forest soils, the gene expression level increases by 4.5 times and the acid phosphatase activity increases by 2.8 times under phosphorus limitation (Chen et al., 2024); while the phytase gene (phy) of *Bacillus* is regulated by PhoB. Under low phosphorus conditions, the expression level is upregulated, the phytase activity is enhanced, and phytic acid salts in soil can be efficiently degraded (Li et al., 2025).

Omics studies have revealed the environmental adaptive regulation of phosphatase production: transcriptome analysis found that the expression profiles of phosphatase genes in PSMs differ significantly between different soil types (acidic vs. alkaline). For example, the expression level of acid phosphatase gene in *Penicillium chrysogenum* in acidic soil is 2.5 times that in alkaline soil, while the opposite is true for alkaline phosphatase gene (Akbar et al., 2023). This difference ensures the organic phosphorus mineralization ability of PSMs in different environments. However, current related studies are mostly focused on the regulation of a single environmental factor, and the regulatory network under combined stresses (e.g., low phosphorus + heavy metals) is not clear.

### 3.3 Synergistic effect of other auxiliary mechanisms

In addition to the core mechanisms, compounds secreted by PSMs such as plant growth regulators (IAA, gibberellins) and siderophores indirectly improve phosphorus absorption efficiency by improving plant root development and regulating rhizosphere microenvironment (Timofeeva et al., 2022; Vasques et al., 2024). For example, *Bacillus* strains promote wheat root branching by secreting IAA, increasing root surface area by more than 30% and phosphorus absorption by 25% (Khan et al., 2009); while siderophores of *Pseudomonas* chelate Fe^3 +^ to release phosphate ions from iron-phosphate complexes, and simultaneously promote plant iron absorption, achieving a synergistic improvement of “phosphorus-iron” nutrition (Thampi et al., 2023). Multi-omics studies have shown that the genes of these auxiliary mechanisms and core phosphorus-solubilizing genes are regulated by the same phosphorus starvation signal, forming a synergistic regulatory network (Bolan et al., 2025). However, the analysis of the interaction between network nodes is still fragmented.

## 4 Environmental regulation of PSMs activity and stress adaptability mechanisms

The phosphorus-solubilizing activity of PSMs is regulated by environmental factors such as soil pH, phosphorus concentration, carbon source, and temperature, among which pH and phosphorus concentration are key regulatory factors. Under stress conditions such as heavy metal pollution, salinity-alkalinity, and drought, PSMs form adaptive mechanisms through their own physiological metabolism adjustment and gene expression remodeling, which is the core premise for their field application.

### 4.1 Regulatory effects of key environmental factors

pH regulates phosphorus-solubilizing efficiency by affecting the metabolic activity of PSMs and the chemical form of insoluble phosphorus. In acidic environments, H^+^ can destroy the lattice structure of calcium phosphate and iron phosphate, and the chelating effect of organic acids is enhanced, significantly improving the dissolution efficiency of inorganic phosphorus (Liu et al., 2023; Pang et al., 2024); in alkaline soils, PSMs need to secrete more organic acids to reduce rhizosphere pH to achieve phosphorus activation, so the screening of high-efficiency PSMs in alkaline soils is more challenging (Naorem et al., 2023). There are differences in the pH adaptation range of different PSM groups: fungi have the best phosphorus-solubilizing efficiency under pH 4.0–6.0, while bacteria are more adaptable to neutral environments with pH 6.5–7.5 (Akbar et al., 2023; Jia et al., 2023). This differentiation is an important driving factor for the community structure of soil PSMs.

Phosphorus concentration regulates PSMs activity through “negative feedback regulation”: under low phosphorus conditions, PSMs activate the phosphorus starvation response system, upregulate the expression of genes related to organic acids and phosphatases, and enhance phosphorus-solubilizing activity (Liu et al., 2023; Richy et al., 2024); under high phosphorus conditions, the phosphorus starvation response system is inhibited, and PSMs do not need to invest energy in synthesizing phosphorus-solubilizing substances, resulting in a significant decrease in phosphorus-solubilizing activity (Zeng et al., 2015). This regulatory mechanism is an evolutionary strategy for PSMs to adapt to environmental phosphorus levels, but it also limits their application effect in high-phosphorus soils.

Carbon source is the energy basis for the metabolic activities of PSMs. Easily utilizable carbon sources (e.g., glucose, sucrose) can significantly increase the secretion of organic acids and phosphatase activity (Liu et al., 2023). The effect of different carbon source types on PSMs activity has species specificity: *Pseudomonas* has the highest phosphorus-solubilizing efficiency when glucose is used as the carbon source, while *Bacillus* prefers sucrose (Wang et al., 2023), which provides a basis for the selection of carbon source additives in PSMs biofertilizers.

### 4.2 Adaptive mechanisms under stress conditions

#### 4.2.1 Adaptability to heavy metal stress

Heavy metals (e.g., Cd, Pb) can damage the cell membrane structure and enzyme activity of PSMs, inhibiting phosphorus-solubilizing function. However, some heavy metal-tolerant PSMs adapt through three mechanisms: first, secreting organic acids to chelate heavy metals and reduce intracellular toxicity (Kumar et al., 2025); second, activating the antioxidant system (e.g., upregulation of superoxide dismutase gene expression) to scavenge reactive oxygen species (Zhu Y. et al., 2024); third, preferentially absorbing phosphorus through the phosphorus transport system to maintain intracellular phosphorus balance (Bolan et al., 2025). For example, in Cd-contaminated soils, *Enterobacter* strains chelate Cd^2+^ by secreting oxalic acid, and simultaneously upregulate the expression of phytase genes to maintain phosphorus-solubilizing activity and promote the remediation of Cd by *Lespedeza bicolor* (Clemensen et al., 2020).

#### 4.2.2 Adaptability to salinity-alkalinity stress

High-salt environments can cause dehydration and osmotic imbalance in PSMs cells. Salt-tolerant PSMs regulate osmotic pressure by accumulating compatible solutes (e.g., proline, betaine), and simultaneously upregulate the expression of salt tolerance-related genes (e.g., Na^+^/H^+^ antiporter gene) to maintain intracellular ion balance (Thampi et al., 2023). For example, salt-tolerant *Bacillus* strains can still dissolve calcium phosphate by secreting gluconic acid under 5% salinity, and the phosphorus-solubilizing efficiency remains above 65% of the normal condition (Xu et al., 2021).

#### 4.2.3 Adaptability to drought stress

Drought inhibits the metabolism and diffusion of PSMs by reducing soil water availability. Drought-adapted PSMs mostly enhance soil water retention by secreting extracellular polymeric substances (EPS), and simultaneously upregulate the expression of drought response genes (e.g., dehydrin gene) to maintain cell activity (Kumar et al., 2025). For example, under drought conditions, the EPS secretion of *Trichoderma* strains increases by 2.1 times, the rhizosphere soil water content increases by 18%, and high phosphatase activity is maintained (Wang et al., 2023).

It is worth noting that existing studies on stress adaptability are mostly focused on single stresses, while field environments are mostly combined stresses (e.g., drought+salinity-alkalinity, heavy metals+low phosphorus). The adaptive mechanisms of PSMs under combined stresses are not clear, which is one of the key reasons for the disconnection between laboratory effects and field applications.

## 5 Host-specific interaction mechanisms between PSMs and plants

The interaction between PSMs and plants is not a generalized relationship but has significant host specificity. This specificity is jointly determined by the composition of plant root exudates, root structure, and the signal recognition system of PSMs, which is a core factor affecting the application effect of PSMs (Bolan et al., 2025; Zhu Y. et al., 2024). Existing studies mostly ignore this characteristic, leading to limited field applicability of PSMs inoculants, which is one of the key innovative directions that distinguish this review from existing ones.

### 5.1 Driving factors of host specificity

The species specificity of plant root exudates is the key driving factor for the host preference of PSMs. There are significant differences in the composition of root exudates (e.g., sugars, amino acids, flavonoids) among different crops: leguminous plants (e.g., soybeans) have high levels of flavonoids in their root exudates, which can specifically induce the chemotaxis and colonization of rhizobia-related PSMs (Vasques et al., 2024); while the root exudates of gramineous plants (e.g., wheat) are mainly glucose and organic acids, which are more likely to attract *Pseudomonas* PSMs (Khan et al., 2024). Transcriptome analysis shows that the expression of chemotaxis factor genes and colonization-related genes (e.g., adhesion protein genes) in PSMs is specific to root exudates, which can only be activated under the induction of exudates from suitable hosts (de Almeida Leite et al., 2024).

Differences in plant root structure also affect interaction efficiency. Fibrous root crops (e.g., wheat) have a large root surface area and more contact sites with PSMs, making it easier to form stable interactions; while taproot crops (e.g., cotton) have a stable rhizosphere microenvironment, which is conducive to the long-term colonization of PSMs (Zhu Y. et al., 2024). In addition, the phosphorus starvation response of plants can also regulate interactions. Under low phosphorus conditions, plants increase the secretion of organic acids in root exudates, further recruiting PSMs colonization (Li et al., 2025).

### 5.2 Functional effects of specific interactions

Host-specific interactions can significantly improve phosphorus absorption efficiency and plant stress resistance. For example, in the specific interaction between soybeans and rhizobia PSMs, PSMs not only dissolve soil phosphorus but also provide nitrogen for soybeans through nitrogen fixation, achieving synergistic supply of “phosphorus-nitrogen,” and soybean yield is increased by 22%–35% (Vasques et al., 2024); in the interaction between wheat and *Pseudomonas* PSMs, siderophores secreted by PSMs can synergistically improve the absorption of phosphorus and iron by wheat, alleviating the “phosphorus-iron” antagonism (Khan et al., 2024). On the contrary, non-specific PSMs inoculation (e.g., inoculating PSMs suitable for leguminous crops directly into gramineous crops) results in a colonization rate of less than 10% and a phosphorus-solubilizing effect of only 25% of specific inoculation (Zhu Y. et al., 2024).

At present, the molecular signaling pathways of host-specific interactions between PSMs and plants are still unclear. Key scientific issues such as how plants recognize PSMs and how PSMs regulate host root development have not been resolved, which need to be further analyzed through combined metatranscriptome and metabolome analysis.

## 6 Agricultural application bottlenecks and solutions of PSMs

**FIGURE 2:**
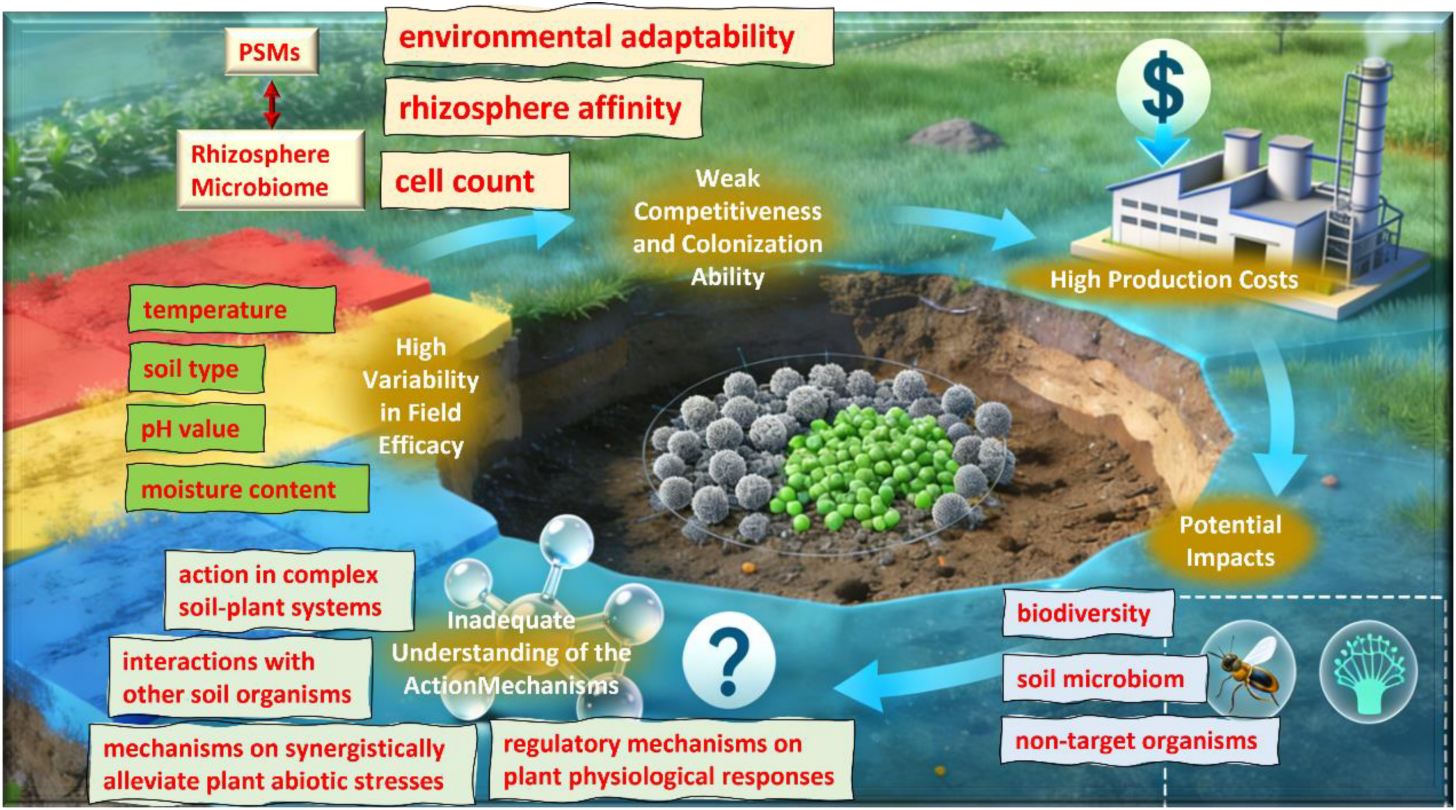
Major bottlenecks in the agricultural application of PSMs (Clemensen et al., 2020; Gyaneshwar et al., 2002; Khan et al., 2024; Raymond et al., 2020; Su L. et al., 2023; Xing et al., 2023)

As a biofertilizer, Phosphate-Solubilizing Microorganisms (PSMs) have great application potential in sustainable agriculture, which can improve the availability of soil phosphorus and promote crop growth (Kaur et al., 2024). However, the agricultural application of PSMs still faces many bottlenecks (Figure 2), requiring comprehensive solutions to overcome.

### 6.1 Optimization of PSMs strain screening and modification

Screen native PSMs strains with excellent phosphorus-solubilizing ability and environmental adaptability from specific crop varieties or agricultural ecosystems (e.g., saline-alkaline soils, oil palm rhizospheres) (Acevedo et al., 2014; Jiang et al., 2018). These native strains may better adapt to local soil environments, improving colonization and competitiveness. Screen and develop PSMs strains tolerant to abiotic stresses such as salinity, heavy metals, drought, and high temperature to improve their application effects in harsh environments (Cao et al., 2023; Lu et al., 2023). For example, studies have found that certain halophilic PSMs (e.g., *Bacillus*) can still effectively solubilize phosphate in saline media (Jiang et al., 2018). Use multi-omics methods such as genomics, proteomics, and metabolomics to deeply analyze the phosphorus-solubilizing mechanisms of PSMs and their interaction mechanisms with plants (Kumar et al., 2023; Lu et al., 2023; Park et al., 2020). Conduct genetic improvement of PSMs through synthetic biology, metabolic engineering, and genetic engineering technologies to enhance their phosphorus-solubilizing efficiency, stress resistance, colonization ability, and ability to secrete plant growth-regulating substances (Lovley and Yao, 2021).

### 6.2 Development of novel carriers and application strategies

Studies have shown that organic materials such as straw compost can be used as effective carriers for PSMs, improving their survival rate and effect in soil (Li C. et al., 2024). Straw compost can improve soil physical and chemical properties, provide a favorable microenvironment for PSMs, thereby promoting the colonization of PSMs in the rhizosphere soil of plants, improving phosphorus-solubilizing efficiency, and further promoting plant phosphorus absorption and growth (Li C. et al., 2024). Use nano/microstructured supramolecular biopolymers as carriers for PSMs to achieve targeted delivery and controlled release of bioactive compounds, improve the stability and bioavailability of PSMs, and improve soil structure and water retention capacity (Saberi Riseh et al., 2024). Combined application of PSMs with other beneficial microorganisms such as Arbuscular Mycorrhizal Fungi (AMF) and Plant Growth-Promoting Microorganisms (PGPM) can exert synergistic effects, more effectively promoting plant growth and nutrient absorption (Cao et al., 2024; Laishram et al., 2024; Sahoo et al., 2024; Trivedi et al., 2021). For example, the combined inoculation of PSMs and AMF can significantly promote the growth of *Phyllostachys edulis* seedlings in phosphorus-deficient soils (Xing et al., 2023).

### 6.3 Optimization of agricultural management measures

Coordinate the application of organic fertilizers, inorganic fertilizers, and PSMs, and implement integrated phosphorus management strategies to improve phosphorus use efficiency, reduce phosphorus fertilizer input and environmental pollution (Bolo et al., 2021; El Attar et al., 2022). For example, improve soil microbial biomass and PSMs abundance by adjusting tillage methods, crop rotation, and residue return (Bolo et al., 2021). Conduct precision fertilization according to soil phosphorus status and crop needs, reduce the use of excessive phosphorus fertilizers, and create a more favorable environment for PSMs to play their roles (El Attar et al., 2022).

### 6.4 In-depth research on the interaction between PSMs and plant secondary metabolites

Plant secondary metabolites play key roles in plant-environment interactions, including responding to abiotic and biotic stresses (Aggarwal et al., 2024; Clemensen et al., 2020, 2025; He et al., 2026; Mandal, 2025; Shelake et al., 2023; Sun and Fernie, 2024). Studying how PSMs regulate plant phosphorus absorption, stress resistance, and interaction with microorganisms by affecting the synthesis and secretion of plant secondary metabolites will help develop more effective PSMs application strategies (Clemensen et al., 2025; Su Y. et al., 2023). Certain plant secondary metabolites have antibacterial activity and can be used to manage antibiotic resistance of bacteria (Kongkham et al., 2020). Combining PSMs (Phosphate-Solubilizing Microorganisms) with biologically active plant secondary metabolites may enhance plant resistance to diseases and insect pests while improving crop productivity.

### 6.5 Strengthening field trials and long-term monitoring

Carry out multi-scale field trial studies to systematically evaluate the long-term effects and environmental safety of PSMs in different ecological regions, soil types, and crop systems (Park et al., 2020; Raymond et al., 2020). Use advanced technologies such as high-throughput sequencing, mass spectrometry, metabolomics, and eco-metabolomics to real-time monitor soil microbial community dynamics, plant metabolic changes, and the fate of PSMs in the soil-plant system (Clemensen et al., 2025; Kumar et al., 2023; Lu et al., 2023; Park et al., 2020), and real-time monitor the potential impacts of PSMs release on the environment and native microbiome biodiversity.

## 7 Discussion and conclusion

Although the research on PSMs has formed a basic framework of “mechanism analysis - application exploration,” combined with industrialization needs and ecosystem complexity, existing research still has deep-seated limitations, and some research paradigms have inherent flaws, which need to be critically examined from the root:

First, the “fragmentation” and “scenario detachment” of molecular mechanism research coexist. Most existing omics studies focus on single strains and are carried out under laboratory pure culture and single environmental factor regulation. Although they can analyze the basic phosphorus-solubilizing pathways of specific strains (e.g., organic acid synthesis genes, phosphorus starvation response systems), they completely separate the multi-dimensional interaction network of “PSMs - plants - soil microorganisms” in soil, resulting in the analyzed molecular mechanisms lacking ecological authenticity. For example, the high-activity phosphorus-solubilizing genes identified in the laboratory may not be normally expressed in the field environment with complex microbial competition and carbon source limitation; while under combined stresses (e.g., low phosphorus + drought + heavy metals), the regulatory network of PSMs is not a simple superposition of single stress pathways, and their synergistic or antagonistic effects have not been systematically analyzed, which directly leads to the disconnection between basic mechanism research and field application needs. In addition, the current understanding of the mechanism differences among different functional groups of PSMs is unbalanced. The research on bacterial mechanisms is relatively sufficient, but the analysis of molecular regulatory pathways of fungi and actinomycetes is lagging behind, especially the phosphorus-solubilizing mechanism of actinomycetes is almost blank, which restricts the comprehensive development of functional resources.

Second, the “superficiality” of host-specific interaction research and the “blindness” of application promotion are contradictory. Although existing studies have confirmed the existence of host specificity between PSMs and plants, the analysis of their driving mechanisms only stays at the superficial description of “differences in root exudate composition,” and does not go deep into the molecular pathway level of “signal recognition - gene regulation - phenotypic response.” For example, the interaction mode between specific signal molecules (e.g., flavonoids, sugars) in plant root exudates and surface receptor proteins of PSMs has not been clarified, nor has it been clarified how PSMs adapt to the rhizosphere microenvironment of different hosts by regulating their own gene expression. This lack of understanding directly leads to the blindness of promoting “broad-spectrum strains” in field applications - most commercial PSMs inoculants do not consider host preference, and directly apply strains suitable for leguminous crops to gramineous crops, resulting in insufficient colonization rate and fluctuating phosphorus-solubilizing effects, which not only causes resource waste but also reduces farmers’ trust in biofertilizers.

Third, the “dichotomous separation” between basic research and practical research hinders the industrialization process. Basic researchers mostly focus on theoretical breakthroughs in molecular mechanisms, ignoring practical bottlenecks such as inoculant stability and application cost; while enterprise R&D focuses on short-term application effects, lacking basic mechanism support, leading to inoculant R&D falling into an inefficient cycle of “screening - verification - elimination.” For example, some high-activity PSMs strains reported in studies cannot achieve large-scale production due to failure to consider fermentation costs and shelf life; while commercial inoculants are difficult to stably play their roles in complex field environments due to lack of in-depth analysis of strain stress resistance mechanisms. In addition, existing research methodologies have inherent flaws. For example, the core method for strain screening - the plate solubilization zone method - can only evaluate the phosphorus-solubilizing potential of strains in high-concentration insoluble phosphorus media, which is significantly different from the actual field environment with low phosphorus and multi-nutrient competition, resulting in “high-efficiency strains” screened with poor field performance, with a correlation of only 0.4–0.6 (Li H. Z. et al., 2024); the lack of long-term field location trials also makes it impossible to evaluate the long-term impact of PSMs on soil microbial community structure and soil fertility evolution, restricting the formulation of sustainable application strategies.

Fourth, the “descriptive bias” of existing reviews lacks critical guidance. Most existing reviews only sort out and summarize the phosphorus-solubilizing mechanisms and application cases of PSMs, without critically analyzing the rationality of research methods and the universality of research conclusions, nor clarifying the priority of research gaps, leading to scattered follow-up research directions and difficulty in forming focused breakthroughs. For example, aiming at the hot direction of “compound microbial inoculant R&D,” existing reviews do not criticize the blindness of single strain compounding, nor propose core evaluation criteria for “functional complementarity” and “interaction compatibility,” leading to the failure of some compound inoculants due to competitive inhibition between strains.

Combined with existing research limitations and the core needs of agricultural green development, future research should take “systematization of basic mechanisms - precision of application technologies - improvement of industrial systems” as the core goals, focus on the following five priority directions, and achieve a full-chain breakthrough from “theoretical breakthrough” to “industrial landing”:

### 7.1 Multi-omics integration + single-cell technology to analyze complex interaction networks

To address the limitation of fragmented molecular mechanisms, it is necessary to break through the research paradigm of “single strain - single environment,” rely on the synergistic advantages of multi-omics technologies, and combine single-cell sequencing technology to achieve panoramic and precise analysis of the ternary interaction system of “PSMs - plants - soil microorganisms.” Specifically, the combination of metagenomics and metatranscriptomics can reveal the species composition, dynamic expression of functional genes, and niche differentiation of microbial communities in the ternary interaction system under different environmental conditions (especially combined stresses), clarifying the competitive/synergistic relationships between PSMs and soil microorganisms; metabolome and secretome analysis can accurately identify key signal molecules mediating interactions (e.g., flavonoids in plant root exudates, organic acids secreted by PSMs), constructing an association network of “genes - metabolites - phenotypes”; single-cell genomics/transcriptomics technology can avoid the “average effect” of metagenomics, accurately locate core functional strains in the interaction network, and analyze their specific regulatory pathways. On this basis, focus on breaking through the synergistic regulatory mechanisms under combined stresses (low phosphorus + heavy metals + drought/salinity-alkalinity), clarify the cross-regulatory nodes between the phosphorus starvation response system and stress resistance system of PSMs, identify core target genes that simultaneously improve phosphorus-solubilizing efficiency and stress resistance, and provide theoretical support for the breeding of high-efficiency stress-tolerant strains. At the same time, it is necessary to establish a mechanism verification system of “laboratory simulation - field validation” to ensure that the analyzed molecular mechanisms have ecological authenticity.

### 7.2 Analysis of molecular signaling pathways of host-specific interactions and precise matching

To address the limitation of superficial host-specific research, it is necessary to focus on the core chain of “signal recognition - gene regulation - colonization adaptation” to clarify the molecular mechanisms of PSMs-plant interactions. First, through combined transcriptome and proteome analysis, identify specific signal molecules in root exudates of different crops (especially major field crops such as corn and soybeans) and corresponding receptor proteins on the surface of PSMs, analyzing the binding mode of signal molecules and receptor proteins and downstream regulatory pathways; second, verify the function of key signaling pathway genes using synthetic biology technology to clarify the molecular basis of PSMs adapting to different hosts; finally, based on mechanism analysis results, establish a precise matching database of “crops - PSMs,” clarifying the dominant PSMs groups suitable for different crops (e.g., rhizobia for leguminous crops, Pseudomonas for gramineous crops), providing theoretical basis for the precise application of inoculants. In addition, it is necessary to carry out specific interaction experiments under different crop-soil type combinations, evaluate the impact of host specificity on PSMs colonization rate and phosphorus-solubilizing effect, and optimize the matching scheme of “crops - strains - soil.”

### 7.3 Gene editing + directed domestication to breed high-efficiency stress-tolerant strains

To address the limitation of poor field adaptability of PSMs, it is necessary to combine the results of molecular mechanism research and adopt a synergistic strategy of “gene editing + directed domestication” to breed high-quality strains with high-efficiency phosphorus solubilization, broad-spectrum stress resistance, and stable colonization ability. On the one hand, based on the core target genes analyzed by the ternary interaction mechanism, use gene editing technologies such as CRISPR-Cas9 to directionally modify PSMs, for example, enhance the expression of core genes in the phosphorus starvation response system (e.g., PhoB) to increase organic acid secretion; introduce heavy metal and salinity-alkalinity tolerance-related genes to enhance adaptability to combined stresses. At the same time, attention should be paid to the ecological safety evaluation of gene-edited strains to avoid damage to the native soil microbial community. On the other hand, carry out long-term directed domestication experiments, domesticate PSMs for multiple generations under simulated field combined stresses (low phosphorus + heavy metals + drought), and screen strains with strong adaptability and stable phosphorus-solubilizing function, making up for the ecological risk shortcomings of gene editing technology. In addition, the development of compound microbial inoculants should take “functional complementarity” and “interaction compatibility” as the core principles, screen functionally complementary strain combinations (e.g., PSMs + nitrogen-fixing bacteria, PSMs + arbuscular mycorrhizal fungi) through *in vitro* interaction experiments, and evaluate the interaction effects between strains using metabolomics to avoid competitive inhibition, achieving a synergistic function of “phosphorus solubilization + stress resistance + growth promotion.”

### 7.4 Long-term field location trials across ecological regions and construction of effect prediction models

To address the disconnection between basic research and practice, it is necessary to establish a three-level verification system of “laboratory simulation - plot experiment - long-term location trial across ecological regions” to systematically evaluate the long-term application effects of PSMs. First, simulate stress environments of different soil types and climatic conditions in the laboratory to evaluate the phosphorus-solubilizing efficiency and stress resistance of PSMs; second, carry out plot experiments in typical ecological regions to optimize the application rate and method (e.g., seed coating, furrow application) of PSMs inoculants; finally, establish long-term location trials in major agricultural ecological regions such as Northeast black soil, North China saline-alkaline soil, and South red soil, continuously monitor the long-term impact of PSMs on crop yield, soil fertility (e.g., soil organic matter content, available phosphorus content), and soil microbial community structure, and evaluate their ecological safety and sustainability. At the same time, combine meteorological data, soil physical and chemical properties, and crop growth indicators to construct a PSMs application effect prediction model using machine learning technology, realizing precise optimization of application strategies under “soil - crop - climate” conditions, and improving the stability of application effects.

### 7.5 Construction of low-cost industrialization technology system and improvement of quality standards

To address industrialization bottlenecks, it is necessary to make breakthroughs in the entire chain of “fermentation process - formulation development - quality control,” reduce application costs, and improve the industrial system. In terms of fermentation process, develop intelligent fermentation technology using agricultural wastes (e.g., corn stover, livestock and poultry manure) as low-cost fermentation substrates, optimize fermentation conditions (temperature, pH, aeration rate), improve strain yield and activity, and reduce fermentation costs; in terms of formulation development, focus on the optimization of carrier materials, develop composite carriers of biochar + trehalose + humic acid, use embedding technology to improve the shelf life and field colonization ability of inoculants, and simplify the application method (e.g., seed coating, foliar spraying) to improve farmers’ acceptance; in terms of quality control, establish a full-chain quality standard system for PSMs biofertilizers, clarify core indicators such as strain activity, purity, shelf life, and heavy metal content, develop rapid detection technologies (e.g., quantitative real-time PCR technology), and realize precise monitoring of product quality. In addition, it is necessary to promote the formulation of international unified quality standards, standardize the market order, and improve the market recognition of PSMs biofertilizers.

In conclusion, as a core biological resource for sustainable phosphorus management, the full exploitation of PSMs application potential depends on the deep integration of basic research and practical research. Future research needs to break through the limitations of existing research paradigms, guide the focus of research directions with critical thinking, and realize the leap of PSMs from “high efficiency in the laboratory” to “stability in the field” through the systematization of mechanism analysis, precision of application technologies, and improvement of industrial systems, providing core support for ensuring food security and promoting the green and sustainable development of agriculture.

## 8 Research methods

Focusing on the core themes of “Phosphate-solubilizing microorganisms (PSMs), Phosphate-solubilizing bacteria (PSB), Phosphate-solubilizing fungi (PSF), Soil phosphorus activation, phosphorus use efficiency, plant-microbe interaction, sustainable agriculture, organic/inorganic insoluble phosphorus, phosphatase, organic acid secretion, environmental factors, biofertilizer, ecological remediation,” this study adopts a systematic literature review approach. The literature retrieval period is restricted to January 2015 to June 2025.

Core SCI-indexed databases were selected, including Web of Science Core Collection, PubMed, SpringerLink, Scopus, ScienceDirect, and CNKI (China National Knowledge Infrastructure), with the latter included to cover high-impact Chinese-language research on PSM applications in agricultural systems. These databases were chosen due to their comprehensive coverage of disciplines related to microbiology, soil science, plant nutrition, agricultural sustainability, and environmental biotechnology. Retrieval terms were decomposed based on the principle of “core concepts + research dimensions,” and the search formula was formulated as: (Phosphate-solubilizing microorganisms OR PSMs OR Phosphate-solubilizing bacteria OR PSB OR Phosphate-solubilizing fungi OR PSF) AND (Soil phosphorus activation OR Phosphorus use efficiency OR Plant-microbe interaction OR Sustainable agriculture). To ensure retrieval precision, the search was restricted to the title and keyword fields.

Initial retrieval using the above strategy yielded 2,863 documents. Subsequently, peer-reviewed papers, reports from authoritative institutions, and data-supported research were adopted as quality criteria. We excluded documents with unreliable translations, non-peer-reviewed literature, studies unrelated to the core dimensions of PSMs, and research lacking policy or technological relevance. Ultimately, 418 high-quality academic documents published in the past 5 years, along with foundational policy documents, were selected as the basis for analysis.

## References

[B1] Acevedo E. Galindo-Castañeda T. Prada F. Navia M. Romero H. M. (2014). Phosphate-solubilizing microorganisms associated with the rhizosphere of oil palm (*Elaeis guineensis* Jacq.) in Colombia. *Appl. Soil Ecol.* 80 26–33. 10.1016/j.apsoil.2014.03.011

[B2] Aggarwal P. R. Mehanathan M. Choudhary P. (2024). Exploring genetics and genomics trends to understand the link between secondary metabolic genes and agronomic traits in cereals under stress. *J. Plant Physiol.* 303:154379. 10.1016/j.jplph.2024.154379 39549316

[B3] Akbar M. Chohan S. A. Yasin N. A. Ahmad A. Akram W. Nazir A. (2023). Mycorrhizal inoculation enhanced tillering in field grown wheat, nutritional enrichment and soil properties. *PeerJ* 11:e15686. 10.7717/peerj.15686 37719109 PMC10504892

[B4] Alori E. T. Glick B. R. Babalola O. O. (2017). Microbial phosphorus solubilization and its potential for use in sustainable agriculture. *Front. Microbiol.* 8:971. 10.3389/fmicb.2017.00971 28626450 PMC5454063

[B5] Amy C. Avice J.-C. Laval K. Bressan M. (2022). Are native phosphate solubilizing bacteria a relevant alternative to mineral fertilizations for crops? Part I. when rhizobacteria meet plant P Requirements. *Rhizosphere* 21:100476. 10.1016/j.rhisph.2022.100476

[B6] Anonymous (2024). Single-cell exploration of the microbiota driving soil phosphorus mobilization. *Nat. Food* 5 654–655. 10.1038/s43016-024-01025-7 39117953

[B7] Bashir Z. Hamid B. Yatoo A. M. Nisa M. Sultan Z. Popescu S. M. (2024). Phosphorus solubilizing microorganisms: An eco-friendly approach for sustainable plant health and bioremediation. *J. Soil Sci. Plant Nutr.* 24 6838–6854. 10.1007/s42729-024-02007-1

[B8] Billah M. Khan M. Bano A. Hassan T. U. Munir A. Gurmani A. R. (2019). Phosphorus and phosphate solubilizing bacteria: Keys for sustainable agriculture. *Geomicrobiol. J.* 36 904–916. 10.1080/01490451.2019.1654043

[B9] Bolan N. Mukherjee S. Sharma S. Bolan S. Yuan J. Yang S. (2025). Exudates of carboxylates by roots and their implications for nutrient, contaminant and carbon dynamics in soil. *Crit. Rev. Plant Sci.* 44 399–421. 10.1080/07352689.2025.2549655

[B10] Bolo P. Kihara J. Mucheru-Muna M. Njeru E. M. Kinyua M. Sommer R. (2021). Application of residue, inorganic fertilizer and lime affect phosphorus solubilizing microorganisms and microbial biomass under different tillage and cropping systems in a ferralsol. *Geoderma* 390:114962. 10.1016/j.geoderma.2021.114962

[B11] Boubekri K. Soumare A. Lyamlouli K. Ouhdouch Y. Hafidi M. Kouisni L. (2023). Improving the efficiency of phosphate rocks combined with phosphate solubilizing actinomycetota to increase wheat growth under alkaline and acidic soils. *Front. Plant Sci.* 14:1154372. 10.3389/fpls.2023.1154372 37235036 PMC10206120

[B12] Campo S. San Segundo B. (2020). Systemic induction of phosphatidylinositol-based signaling in leaves of arbuscular mycorrhizal rice plants. *Sci. Rep.* 10:15896. 10.1038/s41598-020-72985-6 32985595 PMC7522983

[B13] Cao M. Narayanan M. Shi X. Chen X. Li Z. Ma Y. (2023). Optimistic contributions of plant growth-promoting bacteria for sustainable agriculture and climate stress alleviation. *Environ. Res.* 217:114924. 10.1016/j.envres.2022.114924 36471556

[B14] Cao Y. Shen Z. Zhang N. Deng X. Thomashow L. S. Lidbury I. (2024). Phosphorus availability influences disease-suppressive soil microbiome through plant-microbe interactions. *Microbiome* 12:185. 10.1186/s40168-024-01906-w 39342390 PMC11439275

[B15] Chakraborti S. Mandal P. Tiru Z. (2025). Understanding the significance of plant growth-promoting fungi (PGPF) in plant growth and soil health for sustainable agriculture. *Plant and Soil* 10.1007/s11104-025-08017-x

[B16] Chen M. Qin H. Liang Y. Xiao D. Yan P. Yin M. (2024). The phoD-harboring microorganism communities and networks in karst and non-karst forests in Southwest China. *Forests* 15:341. 10.3390/f15020341

[B17] Chen Y. Farooq A. Wei X. Qin L. Wang Y. Zhang L. (2025). Transcriptomic and metabolomic analysis of recalcitrant phosphorus solubilization mechanisms in *Trametes gibbosa*. *Front. Microbiol.* 16:1520459. 10.3389/fmicb.2025.1520459 39967735 PMC11832667

[B18] Cheng Y. Narayanan M. Shi X. Chen X. Li Z. Ma Y. (2023). Phosphate-solubilizing bacteria: Their agroecological function and optimistic application for enhancing agro-productivity. *Sci. Total Environ.* 901:166468. 10.1016/j.scitotenv.2023.166468 37619729

[B19] Clemensen A. K. Provenza F. D. Hendrickson J. R. Grusak M. A. (2020). Ecological implications of plant secondary metabolites - phytochemical diversity can enhance agricultural sustainability. *Front. Sustain. Food Syst.* 4:547826. 10.3389/fsufs.2020.547826

[B20] Clemensen A. K. Uthe H. Sun J. Duke S. E. Liebig M. A. Whippo C. W. (2025). Assessing agroecosystem resilience in annual cropping systems with ecometabolomics. *Agrosyst. Geosci. Environ.* 8:e70092. 10.1002/agg2.70092

[B21] da Silva Rodrigues M. V. de Oliveira J. P. Noriler S. A. Garcia A. B. Pereira U. da Rocha U. N. (2024). Genomic and phylogenetic analysis of plant growth-promoting bacteria. *An. XVII Simpósio Bras. Bioinform.* 2024 131–142. 10.5753/bsb.2024.245593

[B22] Das Mohapatra M. Sahoo R. K. Tuteja N. (2024). Phosphate solubilizing bacteria, *Pseudomonas aeruginosa*, improve the growth and yield of groundnut (*Arachis hypogaea* L.) *Physiol. Mol. Biol. Plants* 30 1099–1111. 10.1007/s12298-024-01478-x 39100873 PMC11291777

[B23] de Almeida Leite R. Martins da Costa E. Cabral Michel D. do Amaral Leite A. de Oliveira-Longatti S. M. de Lima W. (2024). Genomic insights into organic acid production and plant growth promotion by different species of phosphate-solubilizing bacteria. *World J. Microbiol. Biotechnol.* 40:311. 10.1007/s11274-024-04119-3 39198273

[B24] El Attar I. Hnini M. Taha K. Aurag J. (2022). Phosphorus availability and its sustainable use. *J. Soil Sci. Plant Nutr.* 22 5036–5048. 10.1007/s42729-022-00980-z

[B25] Fatima F. Ahmad M. M. Verma S. R. Pathak N. (2021). Relevance of phosphate solubilizing microbes in sustainable crop production: A Review. *Int. J. Environ. Sci. Technol.* 19 9283–9296. 10.1007/s13762-021-03425-9

[B26] Fatima F. Pathak N. Srivastava D. Verma S. R. (2020). Molecular detection and exploration of diversity among fungal consortium involved in phosphate solubilization. *Geomicrobiol. J.* 38 29–35. 10.1080/01490451.2020.1807657

[B27] Fu S. F. Balasubramanian V. K. Chen C. L. Tran T. T. Muthuramalingam J. B. Chou J. Y. (2024). The phosphate-solubilising fungi in sustainable agriculture: Unleashing the potential of fungal biofertilisers for plant growth. *Folia Microbiol.* 69 697–712. 10.1007/s12223-024-01181-0 38937405

[B28] Gao X. Wu G. Yu Y. Chen L. Gao Y. Iqbal K. (2025). Eleven-year nitrogen addition exacerbates phosphorus limitation by reducing plant roots and soil microbial biomass in a temperate Forest. *Soil Ecol. Lett.* 7:250326. 10.1007/s42832-025-0326-y

[B29] Gurbanov R. Kalkanci B. Karadag H. Samgane G. (2021). “Phosphorus solubilizing microorganisms,” in *Biofertilizers*, eds Inamuddin Ahamed M. I. Boddula R. Rezakazemi M. (Hoboken, NJ: Wiley), 151–182.

[B30] Gyaneshwar P. Naresh Kumar G. Parekh L. J. Poole P. S. (2002). Role of soil microorganisms in improving P nutrition of Plants. *Plant Soil* 245 83–93. 10.1023/A:1020663916259

[B31] Haile D. Mekbib F. Assefa F. (2016). Isolation of phosphate solubilizing bacteria from white lupin (*Lupinus albus* L.) rhizosphere soils collected from Gojam, Ethiopia. *J. Fertil. Pestic.* 7:172. 10.4172/2471-2728.1000172

[B32] He S. Wang J. Yang N. Li H. Li K. Li L. (2026). Regulatory mechanisms underlying Stress-induced accumulation of plant secondary metabolites. *J. Appl. Res. Med. Aromat. Plants* 50:100689. 10.1016/j.jarmap.2025.100689

[B33] Iftikhar A. Farooq R. Akhtar M. Khalid H. Hussain N. Ali Q. (2024). Ecological and sustainable implications of phosphorous-solubilizing microorganisms in soil. *Discov. Appl. Sci.* 6:33. 10.1007/s42452-024-05683-x

[B34] Ishfaq M. Wang Y. Xu J. Hassan M. U. Yuan H. Liu L. (2023). Improvement of nutritional quality of food crops with fertilizer: A global meta-analysis. *Agron. Sustain. Dev.* 43:74. 10.1007/s13593-023-00923-7

[B35] Jia X. Wang L. Nussaume L. Yi K. (2023). Cracking the code of plant central phosphate signaling. *Trends Plant Sci.* 28 267–270. 10.1016/j.tplants.2022.12.008 36588035

[B36] Jiang H. Qi P. Wang T. Wang M. Chen M. Chen N. (2018). Isolation and characterization of halotolerant phosphate-solubilizing microorganisms from saline soils. *3 Biotech* 8:461. 10.1007/s13205-018-1485-7 30370202 PMC6204131

[B37] Jiang Y. Tian J. Ge F. (2020). New insight into carboxylic acid metabolisms and pH regulations during insoluble phosphate solubilisation process by *Penicillium oxalicum* PSF-4. *Curr. Microbiol.* 77 4095–4103. 10.1007/s00284-020-02238-2 33063152

[B38] Kaur H. Mir R. A. Hussain S. J. Prasad B. Kumar P. Aloo B. N. (2024). Prospects of phosphate solubilizing microorganisms in sustainable agriculture. *World J. Microbiol. Biotechnol.* 40:291. 10.1007/s11274-024-04086-9 39105959

[B39] Khan A. Jilani G. Akhtar S. Naqvi S. M. S. Rasheed M. H. Khan A. A. (2009). Phosphorus solubilizing bacteria: Occurrence, mechanisms and their role in crop production. *Agric. Food Sci.* 1, 48–58.

[B40] Khan M. S. Zaidi A. Wani P. A. (2007). Role of phosphate-solubilizing microorganisms in sustainable agriculture -A Review. *Agron. Sustain. Dev.* 27 29–43.

[B41] Khan N. Siddiqui M. H. Ahmad S. Ahmad M. M. Siddiqui S. (2024). New insights in enhancing the phosphorus use efficiency using phosphate-solubilizing microorganisms and their role in cropping system. *Geomicrobiol. J.* 41 485–495. 10.1080/01490451.2024.2331111

[B42] Kongkham B. Prabakaran D. Puttaswamy H. (2020). Opportunities and challenges in managing antibiotic resistance in bacteria using plant secondary metabolites. *Fitoterapia* 147:104762. 10.1016/j.fitote.2020.104762 33069839

[B43] Kumar S. Diksha, Sindhu S. S. Kumar R. (2025). Harnessing phosphate-solubilizing microorganisms for mitigation of nutritional and environmental stresses, and sustainable crop production. *Planta* 261:95. 10.1007/s00425-025-04669-2 40131541

[B44] Kumar U. Raj S. Sreenikethanam A. Maddheshiya R. Kumari S. Han S. (2023). Multi-omics approaches in plant–microbe interactions hold enormous promise for sustainable agriculture. *Agronomy* 13:1804. 10.3390/agronomy13071804

[B45] Laishram B. Devi O. R. Dutta R. Senthilkumar T. Goyal G. Paliwal D. K. (2024). Plant-microbe interactions: PGPM as microbial inoculants/biofertilizers for sustaining crop productivity and soil fertility. *Curr. Res. Microb. Sci.* 8:100333. 10.1016/j.crmicr.2024.100333 39835267 PMC11743900

[B46] Lee K.-K. Mok I.-K. Yoon M.-H. Kim H. J. Chung D. (2012). Mechanisms of phosphate solubilization by PSB (phosphate-solubilizing bacteria) in soil. *Korean J. Soil Sci. Fertil.* 45 169–176. 10.7745/KJSSF.2012.45.2.169

[B47] Li C. Zheng Z. Zhao Y. Wang H. Li P. Xu J. (2024). Phosphate-solubilizing microorganisms stimulate physiological responses of perennial ryegrass to phosphorus deficiency with assistance of straw compost. *Agronomy* 14:1008. 10.3390/agronomy14051008

[B48] Li H. Z. Peng J. Yang K. Zhang Y. Chen Q. L. Zhu Y. G. (2024). Single-cell exploration of active phosphate-solubilizing bacteria across diverse soil matrices for sustainable phosphorus management. *Nat. Food* 5 673–683. 10.1038/s43016-024-01024-8 39103543

[B49] Li X.-L. Sun H. Zhou J. Chen Y. Du H. Q. Ming Y. X. (2025). Acidification associated with plant phosphorus-acquisition strategies decreases nutrient cycling potential of rhizosphere bacteria along the Hailuogou post-glacial chronosequence. *Plant Soil* 10.1007/s11104-025-07445-z

[B50] Li Y. Wang J. He L. Xu X. Wang J. Ren C. (2022). Different mechanisms driving increasing abundance of microbial phosphorus cycling gene groups along an elevational gradient. *iScience* 25:105170. 10.1016/j.isci.2022.105170 36204265 PMC9529982

[B51] Li Z. Wang Y. Liu Z. Han F. Chen S. Zhou W. (2023). Integrated application of phosphorus-accumulating bacteria and phosphorus-solubilizing bacteria to achieve sustainable phosphorus management in saline soils. *Sci. Total Environ.* 885:163971. 10.1016/j.scitotenv.2023.163971 37150466

[B52] Liu F. Qian J. Zhu Y. Wang P. Hu J. Lu B. (2023). Phosphate solubilizing microorganisms increase soil phosphorus availability: A review. *Geomicrobiol. J.* 41 1–16. 10.1080/01490451.2023.2272620

[B53] Lobo C. B. Juárez Tomás M. S. Viruel E. Ferrero M. A. Lucca M. E. (2019). Development of low-cost formulations of plant growth-promoting bacteria to be used as inoculants in beneficial agricultural technologies. *Microbiol. Res.* 219 12–25. 10.1016/j.micres.2018.10.012 30642462

[B54] Lopez A. Anzuay M. S. Loser U. A. Taurian T. Furlan A. L. (2025). The combined effects of drought stress and phosphorus deficiency on peanut (*Arachis hypogaea* L.) plants are mitigated by drought tolerant phosphate-solubilizing bacteria. *Symbiosis* 97 53–70. 10.1007/s13199-025-01080-z

[B55] Lovley D. R. Yao J. (2021). Intrinsically conductive microbial nanowires for ‘green’ electronics with novel functions. *Trends Biotechnol.* 39 940–952. 10.1016/j.tibtech.2020.12.005 33419586

[B56] Lü J. Gao X. Dong Z. An L. (2011). Expression of mitochondrial malate dehydrogenase in *Escherichia coli* improves phosphate solubilization. *Ann. Microbiol.* 62 607–614. 10.1007/s13213-011-0297-3

[B57] Lu L. Qin W. Wu M. Chen Q. Pan B. Xing B. (2025). Biochar promotes FePO4 solubilization through modulating organic acids excreted by *Talaromyces pinophilus*. *Carbon Res.* 4:27. 10.1007/s44246-025-00193-w

[B58] Lu Z. He S. Kashif M. Zhang Z. Mo S. Su G. (2023). Effect of ammonium stress on phosphorus solubilization of a novel marine mangrove microorganism *Bacillus aryabhattai* NM1-A2 as revealed by integrated omics analysis. *BMC Genomics* 24:550. 10.1186/s12864-023-09559-z 37723472 PMC10506230

[B59] Mahdi S. S. (2012). Soil phosphorus fixation chemistry and role of phosphate solubilizing bacteria in enhancing its efficiency for sustainable cropping - A review. *J. Pure Appl. Microbiol.* 66 1905–1911.

[B60] Mandal S. (2025). Shaping the plant specialized metabolites through modern breeding technique. *Mol. Biotechnol.* 10.1007/s12033-025-01455-z [Epub ahead of print]. 40506580

[B61] Naorem A. Jayaraman S. Dang Y. P. Dalal R. C. Sinha N. K. Rao C. S. (2023). Soil constraints in an arid environment-challenges, prospects, and implications. *Agronomy* 13:220. 10.3390/agronomy13010220

[B62] Pang F. Li Q. Solanki M. K. Wang Z. Xing Y. X. Dong D. F. (2024). Soil phosphorus transformation and plant uptake driven by phosphate-solubilizing microorganisms. *Front. Microbiol.* 15:1383813. 10.3389/fmicb.2024.1383813 38601943 PMC11005474

[B63] Park S. Y. Ufondu A. Lee K. Jayaraman A. (2020). Emerging computational tools and models for studying gut microbiota composition and function. *Curr. Opin. Biotechnol.* 66 301–311. 10.1016/j.copbio.2020.10.005 33248408 PMC7744364

[B64] Pfeifer L. Helming K. Schneider H. Ewert F. (2024). Impact mapping tool for interdisciplinary research institutes. *Soc. Impacts* 3:100048. 10.1016/j.socimp.2024.100048

[B65] Rawat P. Das S. Shankhdhar D. Shankhdhar S. C. (2020). Phosphate-solubilizing microorganisms: Mechanism and their role in phosphate solubilization and uptake. *J. Soil Sci. Plant Nutr.* 21 49–68. 10.1007/s42729-020-00342-7

[B66] Raymond N. S. Gómez-Muñoz B. van der Bom F. J. T. Nybroe O. Jensen L. S. Müller-Stöver D. S. (2020). Phosphate-solubilising microorganisms for improved crop productivity: A critical assessment. *New Phytol.* 229 1268–1277. 10.1111/nph.16924 32929739

[B67] Richy E. Fort T. Odriozola I. Kohout P. Barbi F. Martinovic T. (2024). Phosphorus limitation promotes soil carbon storage in a boreal forest exposed to long-term nitrogen fertilization. *Glob. Chang. Biol.* 30:e17516. 10.1111/gcb.17516 39311643

[B68] Rodríguez H. Fraga R. (1999). Phosphate solubilizing bacteria and their role in plant growth promotion. *Biotechnol. Adv.* 17 319–339. 10.1016/s0734-9750(99)00014-2 14538133

[B69] Saberi Riseh R. Hassanisaadi M. Vatankhah M. Varma R. S. Thakur V. K. (2024). Nano/micro-structural supramolecular biopolymers: Innovative networks with the boundless potential in sustainable agriculture. *Nanomicro Lett.* 16:147. 10.1007/s40820-024-01348-x 38457088 PMC10923760

[B70] Sahoo S. Panda S. S. Kumar S. Nigam R. Sarangi S. Choudhary M. (2024). A review on plant-microbe interactions and its defence mechanism. *Plant Cell Biotechnol. Mol. Biol.* 25 159–175. 10.56557/pcbmb/2024/v25i11-128920

[B71] Salsabila N. Fitriatin B. N. Hindersah R. (2023). The role of phosphate-solubilizing microorganisms in soil health and phosphorus cycle: A review. *Int. J. Life Sci. Agric. Res.* 2 281–287. 10.55677/ijlsar/V02I09Y2023-02

[B72] Šeremešić S. Tančić Živanov S. Rajković M. Aćin V. Milić S. Babec B. (2024). Exploring fungal biodiversity in crop rotation systems: Impact of soil fertility and winter wheat cropping. *Plants* 14:65. 10.3390/plants14010065 39795325 PMC11722751

[B73] Sharma S. Kumari N. Prasad B. (2021). Phosphate-solubilising microorganisms as potential biofertilizer: A review. *Agric. Rev.* 44 84–88. 10.18805/ag.R-2110

[B74] Sharon J. A. Hathwaik L. T. Glenn G. M. (2016). Isolation of efficient phosphate solubilizing bacteria capable of enhancing tomato plant growth. *J. Soil Sci. Plant Nutr*. 10.4067/s0718-95162016005000043 27315006

[B75] Shelake R. M. Jadhav A. M. Bhosale P. B. Kim J. Y. (2023). Unlocking secrets of nature’s chemists: Potential of CRISPR/Cas-based tools in plant metabolic engineering for customized nutraceutical and medicinal profiles. *Plant Physiol. Biochem.* 203:108070. 10.1016/j.plaphy.2023.108070 37816270

[B76] Silva L. Pereira M. C. Carvalho A. Buttrós V. H. Pasqual M. Dória J. (2023). Phosphorus-solubilizing microorganisms: A key to sustainable agriculture. *Agriculture* 13:462. 10.3390/agriculture13020462

[B77] Sivasakthivelan P. Saranraj P. Al Tawaha A. R. M. Arivukkarasu K. Imran Amanullah. (2021). Phosphate solubilizing bacteria and its role in plant growth enhancement: A review. *Adv. Environ. Biol.* 15 1–8.

[B78] Somtrakoon K. Chouychai W. (2021). *Phosphorus Deficiency in Plant and Roles of Phosphate-Solubilizing Bacteria.*

[B79] Su L. Zhang T. Wu M. Zhong Y. Cheng Z. M. (2023). Transcriptome and metabolome reveal sugar and organic acid accumulation in *Rosa roxburghii* fruit. *Plants* 12:3036. 10.3390/plants12173036 37687283 PMC10490343

[B80] Su Y. Wang J. Gao W. Wang R. Yang W. Zhang H. (2023). Dynamic metabolites: A bridge between plants and microbes. *Sci. Total Environ.* 899:165612. 10.1016/j.scitotenv.2023.165612 37478935

[B81] Sun Y. Fernie A. R. (2024). Plant secondary metabolism in a fluctuating world: Climate change perspectives. *Trends Plant Sci.* 29 560–571. 10.1016/j.tplants.2023.11.008 38042677

[B82] Thampi M. Dhanraj N. D. Prasad A. Ganga G. Jisha M. S. (2023). Phosphorus Solubilizing Microbes (PSM): Biological tool to combat salinity stress in crops. *Symbiosis* 91 15–32. 10.1007/s13199-023-00947-3

[B83] Timofeeva A. Galyamova M. Sedykh S. (2022). Prospects for using phosphate-solubilizing microorganisms as natural fertilizers in agriculture. *Plants* 11:2119. 10.3390/plants11162119 36015422 PMC9414882

[B84] Trivedi P. Mattupalli C. Eversole K. Leach J. E. (2021). Enabling sustainable agriculture through understanding and enhancement of microbiomes. *New Phytol.* 230 2129–2147. 10.1111/nph.17319 33657660

[B85] Vasques N. C. Nogueira M. A. Hungria M. (2024). Increasing application of multifunctional *Bacillus* for biocontrol of pests and diseases and plant growth promotion: Lessons from Brazil. *Agronomy* 14:1654. 10.3390/agronomy14081654

[B86] Wang C. Pan G. Lu X. Qi W. (2023). Phosphorus solubilizing microorganisms: Potential promoters of agricultural and environmental engineering. *Front. Bioeng. Biotechnol.* 11:1181078. 10.3389/fbioe.2023.1181078 37251561 PMC10213388

[B87] Wang S. Li Y. Zhang J. Wang X. Hong J. Qiu C. (2022). Transcriptome profiling analysis of phosphate-solubilizing mechanism of *Pseudomonas* strain W134. *Microorganisms* 10:1998. 10.3390/microorganisms10101998 36296274 PMC9609647

[B88] Wang X. Li Z. Li Q. Hu Z. (2025). Alleviation of plant abiotic stress: Mechanistic insights into emerging applications of phosphate-solubilizing microorganisms in agriculture. *Plants* 14:1558. 10.3390/plants14101558 40431124 PMC12115179

[B89] Wang Y. Zhou J. Liu X. Hao L. Yang Y. Yang S. (2025). Organic acid accumulation pattern and its key genes in Chinese cherry fruits. *BMC Genomics* 26:774. 10.1186/s12864-025-11969-0 40855411 PMC12376409

[B90] Wu F. Li J. Chen Y. Zhang L. Zhang Y. Wang S. (2019). Effects of phosphate solubilizing bacteria on the growth, photosynthesis, and nutrient uptake of camellia oleifera abel. *Forests* 10:348. 10.3390/f10040348

[B91] Xiao D. He X. Zhang W. Hu P. Sun M. Wang K. (2022). Comparison of bacterial and fungal diversity and network connectivity in karst and non-karst forests in southwest China. *Sci. Total. Environ.* 822:153179. 10.1016/j.scitotenv.2022.153179 35051465

[B92] Xing Y. Wang F. Yu S. Zhu Y. Ying Y. Shi W. (2023). Enhancing *Phyllostachys edulis* seedling growth in phosphorus-deficient soil: Complementing the role of phosphate-solubilizing microorganisms with arbuscular mycorrhizal fungi. *Plant Soil* 497 449–466. 10.1007/s11104-023-06406-8

[B93] Xu J. Gao W. Zhao B. Chen M. Ma L. Jia Z. (2021). Bacterial community composition and assembly along a natural sodicity/salinity gradient in surface and subsurface soils. *Appl. Soil Ecol.* 157:103731. 10.1016/j.apsoil.2020.103731

[B94] Xu T. Chen P. (2025). Isolation and characterization of phosphate solubilizing bacteria from phosphate tailing soil and their lead passivation potential. *Geomicrobiol. J.* 42 610–618. 10.1080/01490451.2025.2501335

[B95] Yang T. Li L. Wang B. Tian J. Shi F. Zhang S. (2022). Isolation, mutagenesis, and organic acid secretion of a highly efficient phosphate-solubilizing fungus. *Front. Microbiol.* 13:793122. 10.3389/fmicb.2022.793122 35547144 PMC9082945

[B96] Zeng Q. Peñuelas J. Sardans J. Zhang Q. Zhou J. Yue K. (2024). Keystone bacterial functional module activates P-mineralizing genes to enhance enzymatic hydrolysis of organic P in a subtropical forest soil with 5-year N addition. *Soil Biol. Biochem.* 192:109383. 10.1016/j.soilbio.2024.109383

[B97] Zeng Q. Wu X. Wen X. (2015). Effects of soluble phosphate on phosphate-solubilizing characteristics and expression of gcd gene in *Pseudomonas frederiksbergensis* JW-SD2. *Curr. Microbiol.* 72 198–206. 10.1007/s00284-015-0938-z 26573634

[B98] Zhu Y. Xing Y. Li Y. Jia J. Ying Y. Shi W. (2024). The role of phosphate-solubilizing microbial interactions in phosphorus activation and utilization in plant-soil systems: A review. *Plants* 13:2686. 10.3390/plants13192686 39409556 PMC11478493

[B99] Zhu Y.-G. Li H.-Z. Peng J. Yang K. Zhang Y. Y. Chen Q. L. (2024). *Single-Cell Exploration of Active Microbiota in Solubilizing Fixed Phosphorus in Soils.* Berlin: Springer Science and Business Media LLC. 10.21203/rs.3.rs-3931032/v1

